# Loss of pseudo-momentum, energy-release rate and the effective mass of a moving dislocation

**DOI:** 10.1098/rsta.2023.0232

**Published:** 2023-12-25

**Authors:** Xanthippi Markenscoff

**Affiliations:** Department of Mechanical and Aerospace Engineering, University of California, San Diego, La Jolla, CA 92093-0411, USA

**Keywords:** dislocation dynamics, quasi-momentum, energy-release rate, multi-scale mechanics

## Abstract

In a seminal paper in the Philosophical Transactions of the Royal Society (A244, 87–112). Eshelby (Eshelby 1951 *Phil. Trans. R. Soc. Lond. A* 244, 87–112. (doi:10.1098/rsta.1951.0016)) introduced the concept of ‘the force on an elastic singularity’ and suggested that the extensions to dynamics include the application of the momentum flux. In this direction, it is shown that, in the non-uniformly motion of a dislocation there is a loss in quasi-momentum (or pseudo-momentum) across the scales ε to ε^2^, which induces an effective mass of the dislocation, and a loss in kinetic energy across the scales. It is shown through Noether's theorem that the rate of change of quasi-momentum in the volume enclosing the dislocation is equal to the flux of it through the surface minus a quasi-force, which is the dynamic *J* integral. The connection between the variation in the Hamiltonian and in the Lagrangian relates the quasi-force to the energy-release rate, yielding the same effective mass, while providing physical meaning both through momentum and energy. The effective mass of a dislocation is important in relating the energetics of defects from the micro to the macro scales, and the loss of quasi-momentum can have wider applications in continua.

This article is part of the theme issue 'Foundational issues, analysis and geometry in continuum mechanics'.

## 1. Introduction

**Figure 1.**
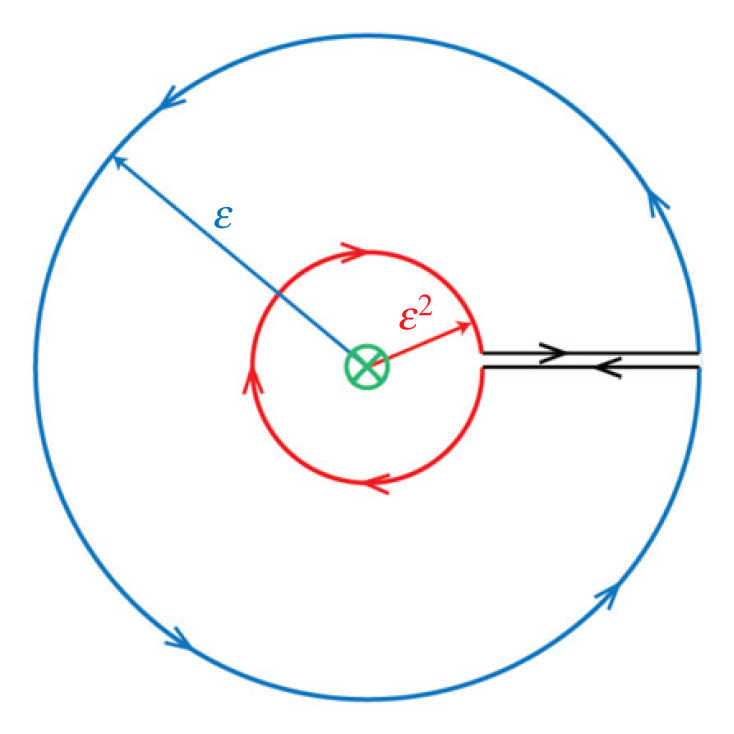
In multi-scale contour across the scales ε to ε^2^ there is a loss of quasi-momentum, with the loss inducing an effective force, and, in turn, an ‘effective mass’ associated with the acceleration of the dislocation. There is a corresponding loss to the kinetic energy flux in the direction of the dislocation motion.

In a seminal paper in the Philosophical Transactions of the Royal Society, Eshelby [1] introduced the concept of ‘the force on an elastic singularity’ in an analogy to the ‘Maxwell tensor’ and proposed extensions to dynamics through the energy release rate, but also suggested to include the application of the momentum flux. Here the singularity concerns a moving dislocation (screw). Linear theory of elasticity is insufficient to determine the motion of a singularity when the velocity of the singular point is not uniform. Eshelby [1] proposed the condition that the energy flux through a small sphere moving with the dislocation be balanced by applied stresses, and suggested that ‘we should have to try to interpret the momentum flux and apply a similar argument to it’. This is accomplished here. It is shown by application of Noether's theorem to a multi-scale contour (figure 1) that the loss of quasi-momentum across scales (ε to ε^2^) induces an effective mass to a moving dislocation associated with the acceleration. Through Noether's [2] theorem, the connection is made between the variation in the Lagrangian and the Hamiltonian in an infinitesimal translation of the defect position, showing that the effective mass as obtained from the quasi-momentum and the energy-release rate (the energy spent per unit advancement if the defect) coincide. It is shown that the rate of change of quasi-momentum in the volume is equal to the flux of it through the surface surrounding the defect minus a quasi-force associated with the energy release rate, which is shown to involve the same effective mass. The effective mass of a dislocation was obtained to have a ${\left(1-v_{d}^{2}/c_{2}^{2}\right)}^{\left(-3/2\right)}$ dependence on the ratio of the dislocation velocity *v^d^* to the shear wave speed *c*_2_.

As macroscopic dynamic plasticity is the effect on the micro-scale of processes occurring at the micro-scale, [3] calculated the elastic precursor decay of a plastic wave from the energy lost to accelerate the dislocations. In the literature, the effective mass was classically treated based on uniform motion of dislocations (e.g. [4,5]), although Eshelby [6] considered an accelerating motion containing a logarithmic singularity. The issue has received attention, but was never solved satisfactorily (e.g. [7]).

## 2. Loss of quasi-momentum across scales (*ε* to *ε*^2^) and effective mass of an accelerating dislocation

The kinetics of defects is governed by Noether's [2] theorem of the calculus of variations in a variable domain (for application to elastic solids, e.g. [8,9]). We consider the Lagrangian functional

2.1 $$\Pi^{L}=\int_{0}^{t}\text{d}t^{\prime }\int_{\Omega }L(u_{i,j}{\dot{u}}_{i})\;\text{d}V=\int_{0}^{t}\text{d}t^{\prime }\int_{\Omega }(T-W)\;\text{d}V,$$

where *u_i_*(*x_j_*, *t*) is the displacement, $T=(1/2)\rho {\dot{u}}_{i}{\dot{u}}_{i}$ is the kinetic energy density and *W* = (1/2)*C_ijkl_u_i_*_,*j*_*u_k_*_,*l*_ is the strain energy density for an elastic solid.

We apply Noether's theorem [2] to the invariance of the functional in (1) under a group of transformations in infinitesimal translation ([10], also [11]). For translation of the inhomogeneity, we use the transformation such that the new coordinates are $x_{i}^{*}=x_{i}+\varepsilon a_{i}$ where ε*a_i_* is the infinitesimal translation of the defect, and t* = *t*, $u_{i}^{*}=u_{i}$ . Then, for invariance of the Lagrangean functional in equation (2.1) under the translations ε*a_k_*, it is obtained

2.2 $$\delta \Pi^{L}=\varepsilon a_{k}\int_{0}^{t}\text{d}t^{\prime }\int_{R}\left\{\frac{\partial }{\partial x_{i}}\{(T-W)\delta_{ik}+\sigma_{ij}u_{j,k}\}-\frac{\partial }{\partial t}(\rho {\dot{u}}_{j}u_{j,k})\right\}\text{d}V=0,$$

where the Euler–Lagrange equations of conservation of linear momentum were assumed to be satisfied.

For this to be valid at any arbitrary time and for any arbitrary volume, the conservation law is obtained

2.3 $$\frac{\partial }{\partial x_{i}}\{(T-W)\delta_{ik}+\sigma_{ij}u_{j,k}\}-\frac{\partial }{\partial t}(\rho {\dot{u}}_{j}u_{j,k})=0,$$

and valid in the region of analyticity. The second term is called pseudo-momentum (e.g. [8]), or quasi-momentum [12]. It should be noted that equation (2.3) is an independent field equation. As such it may be used for testing the accuracy of the numerical solution of the momentum equations (proposed by Maugin).

A generally moving screw dislocation, with motion *x* = *l*(*t*), satisfies the equation of linear momentum $\partial^{2}u_{y}^{\;}/\partial x^{2}+\partial^{2}u_{y}^{\;}/\partial z^{2}=1/c_{2}^{2}\partial^{2}u_{y}/\partial t^{2}$, with boundary condition *u_y_*(*x*, 0^+^, *t*) − *u_y_*(*x*, 0^−^, *t*) = (1/2)*b_y_H*(*x* − *l*(*t*)) where *b_y_* is the Burgers vector, and with initial condition starting from rest [13]. The fields of a generally moving dislocation are obtained as integrals over the history of the motion *x* = *l*(*t*) as the integral of wavelets emitted by the dislocation over the course of its motion [13]. The asymptotic fields near the core are *O*(1/*r*), to the leading order, and are those of the steady-state motion (e.g. [3,4]). For motion with acceleration $\ddot{l}(t)$, there are logarithmically singular terms. The introduction of the acceleration introduces a characteristic length scale ($\ddot{l}/c_{2}^{2}$), so that a logarithmic term can exist (in a dimensionless distance from the core). The order *O*(lnε) term in the displacement gradient $\partial u_{y}/\partial z$ was obtained by singular asymptotic of integrals in Callias & Markenscoff [14] as,

2.4 $$\frac{b_{y}\ddot{l}(t)}{4\pi c_{2}^{2}\gamma^{3}}\ln\varepsilon ,$$

where $\gamma ={\left(1-{\dot{l}}^{2}/c_{2}^{2}\right)}^{1/2}$, and with the superscript (log) denoting the logarithmic part. Thus, at a near-field point *x* = *l*(*t*) + εcos *θ*, *z* = εsin *θ*, we have

2.5a $$\frac{\partial u_{y}}{\partial z}=\frac{b_{y}}{2\pi }\frac{\gamma \cos\;\theta }{\cos^{2}\;\theta +\gamma^{2}\sin^{2}\;\theta }\frac{1}{\varepsilon }+\frac{b_{y}\ddot{l}(t)}{4\pi c_{2}^{2}\gamma^{3}}\ln\varepsilon +O(1),$$

2.5b $$\frac{\partial u_{y}}{\partial x}=-\frac{b_{y}}{2\pi }\frac{\gamma \sin\;\theta }{\cos^{2}\;\theta +\gamma^{2}\sin^{2}\;\theta }\frac{1}{\varepsilon }+O(1),$$

2.5c $$\text{and}\;\frac{\partial u_{y}}{\partial t}=\frac{b_{y}}{2\pi }\frac{\dot{l}(t)\gamma \sin\theta }{\cos^{2}\;\theta +\gamma^{2}\sin^{2}\;\theta }\frac{1}{\varepsilon }+O(1).$$

Ni & Markenscoff [15] showed that $\frac{\partial u_{y}}{\partial x}$ and $\frac{\partial u_{y}}{\partial t}$ do not contain logarithmic terms if singularities of *O*(lnε/ε) are excluded in the governing equation.

We will integrate (using MATHEMATICA) the pseudo-momentum $-(\partial /\partial t)(\rho {\dot{u}}_{j}u_{j,k})$ in a multi-scale contour from radius ε^2^ to ε per unit thickness in the *y*-direction (figure 1)

2.6 $$\begin{array}{rl} -\int_{V(\varepsilon^{2},\varepsilon )}\frac{\partial }{\partial t}(\rho {\dot{u}}_{j}u_{j,1})\;\text{d}V & \;=-\int_{\varepsilon^{2}}^{\varepsilon }\int_{0}^{2\pi }\frac{\partial }{\partial t}(\rho {\dot{u}}_{2}u_{2,1})r\;\text{d}r\;\text{d}\theta \\ & \;=\int_{\varepsilon^{2}}^{\varepsilon }\int_{0}^{2\pi }\frac{\partial }{\partial t}\left\{\rho \frac{b_{y}^{2}}{4\pi^{2}}\frac{\dot{l}(t)\gamma^{2}\sin^{2}\;\theta }{{\left(\cos^{2}\;\theta +\gamma^{2}\text{si}{\text{n}}^{2}\theta \right)}^{2}r^{2}}\right\}r\;\text{d}r\;\text{d}\theta \\ & \;=\frac{\partial }{\partial t}\left\{\frac{\rho b_{y}^{2}}{4\pi^{2}}\frac{\pi \dot{l}(t)}{\gamma^{3}}\right\}[\ln\varepsilon -\ln\varepsilon^{2}]=-\frac{\rho b_{y}^{2}}{4\pi }\frac{\ddot{l}(t)}{\gamma^{3}}\ln\varepsilon , \end{array}$$

which is due to the fact that the dislocation is 1/*r* singular at the core. It may be noted that the same result is obtained for transition between scales (ε*^n^*^−1^, ε*^n^*, *n* > 2); if it is across multiple orders then the effective mass is increased accordingly.

It can be shown by calculation that the value of change of pseudo-momentum in the volume obtained in equation (2.6) agrees with the flux of the pseudo-momentum through the surface of the contour in figure 1, consistent with the conservation equation (2.3), as the contour does not contain a singularity.

We note that contribution to equation (2.6) is only due to the terms in equations (2.5*b*) and (2.5*c*), not (2.5*a*).

Thus, the coefficient of the acceleration can be defined as the effective mass of a dislocation,

2.7 $$m^{\text{eff}}=\frac{\rho b_{y}^{2}}{4\pi \gamma^{3}}\ln\varepsilon \equiv \frac{\mu b_{y}^{2}}{4\pi c_{2}^{2}\gamma^{3}}\ln\varepsilon ,$$

with the negative sign in equation (2.6) meaning that an external force needs to be supplied to accelerate the dislocation. The small scale ε in the effective mass has to be obtained from matching with atomistics.

We can relate the quasi-momentum to the kinetic energy flux when considering a ‘rigid motion’ [12], *x* = *l*(*t*), so that near the moving dislocation the derivatives are related as $\partial /\partial t=-\dot{l}\partial /\partial x$. Then, it is easily seen that identically

2.8 $$-\frac{\partial }{\partial t}(\rho {\dot{u}}_{j}u_{j,k})=-2\frac{\partial T}{\partial x_{k}}.$$

If we integrate equation (2.8) in the volume from radius (ε^2^ to ε) per unit thickness in the *y*-direction (figure 1), we obtain the difference in the flux of kinetic energy density in the direction of the motion as

2.9 $$2\int_{\varepsilon }T\text{d}S_{1}-2\int_{\varepsilon^{2}}T\text{d}S_{1}=\int_{V(\varepsilon^{2},\varepsilon )}\frac{\partial }{\partial t}(\rho {\dot{u}}_{j}u_{j,1})\;\text{d}V=\frac{\rho b_{y}^{2}}{4\pi }\frac{\ddot{l}(t)}{\gamma^{3}}\ln\varepsilon ,$$

which is a positive quantity signifying loss of kinetic energy in the direction of the dislocation motion across scales as the accelerating core extracts energy from the bulk.

## 3. Loss of quasi-momentum, energy-release rate and effective mass of the dislocation

We will now define the dynamic *J* integral as the difference of production of quasi-momentum in a volume minus the flux of it through the surrounding surface. If the volume does not contain a singularity, the two are equal according to equation (2.3). However, if a singularity is enclosed, this creates a negative quasi-force, which will be related here to the energy-release rate (in a ‘metaphysical way’(!), [12]).

**Figure 2.**
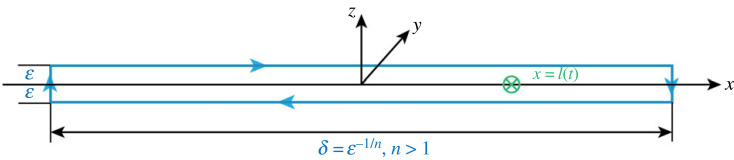
A screw dislocation is moving non-uniformly according to *x* = *l(t);* the energy-release rate is evaluated through the contour in blue as ε → 0, *δ* = ε^−1/*n*^ → ∞, (for *n* > 1), and the volume shrinks to zero.

We integrate on a contour *S* surrounding the dislocation and shrinking onto it in a vanishingly small volume (figure 2), and define the dynamic *J* integral as

3.1 $$\begin{array}{rl} J_{k}^{\text{dyn}} & \;=-\int_{\Omega }\left\{\frac{\partial }{\partial x_{i}}[(T-W)\delta_{ik}+\sigma_{ij}u_{j,k}]-\frac{\partial }{\partial t}(\rho {\dot{u}}_{j}u_{j,k})\right\}\text{d}V\\ & \;=\int_{\Omega }\left\{\frac{\partial }{\partial x_{i}}[(W-T)\delta_{ik}-\sigma_{ij}u_{j,k}]+\frac{\partial }{\partial t}(\rho {\dot{u}}_{j}u_{j,k})\right\}\text{d}V\\ & \;=\int_{S}[(W+T)n_{k}-\sigma_{ij}u_{j,k}n_{i}]\text{d}S+\int_{\Omega }\left(\rho {\ddot{u}}_{j}u_{j,k}-\rho {\dot{u}}_{j}{\dot{u}}_{j,k}\right)\;\text{d}V \end{array}$$

On account of equation (2.8) the last integral in (3.1) vanishes, while, as shown in Markenscoff & Singh [11], the variation of the Hamiltonian functional

$$\Pi^{H}=\int_{0}^{t}\text{d}t^{\prime }\int_{\Omega }(T+W)\;\text{d}V,$$

in an infinitesimal translation ε*a_k_* is

3.2 $$\delta \Pi^{H}=\varepsilon a_{k}\int_{0}^{t}\text{d}t^{\prime }\int_{R}\frac{\partial }{\partial x_{i}}\{(T+W)\delta_{ik}-\sigma_{ij}u_{j,k}\}\;\text{d}V,$$

and is equal to minus the variation of the Lagrangian (expressed in equation (3.1)).

With the velocity of the defect being $\upsilon_{k}=\varepsilon {\dot{a}}_{k}$, and after taking the time derivative of (3.2), we obtain the energy-release rate, that is the rate at which energy passes through the surface *S_d_* toward the core of the dislocation (for cracks, [16,17]), as

3.3 $$\delta {\dot{\Pi }}^{H}=\int_{S_{d}\to 0}\left\{(T+W)\upsilon_{n}+\sigma_{ij}{\dot{u}}_{j}n_{i}\right\}\;\text{d}S,$$

where υ*_n_* is the component of the velocity of the defect in the direction of the outward normal to the surface *S_d_*. We evaluate the integral in (3.3) for the contour shown in figure 2, in the limit as the parameter ε → 0, *δ* = ε^−1/*n*^ → ∞, (for *n* > 1), and the volume shrinks to zero.

For dislocations moving from rest to a constant velocity, the limit of the integral in (3.3) was shown in Clifton & Markenscoff [3] to be independent of the shape of the contour as it shrinks onto the dislocation. The contributing term of equation (3.3), when the dislocation velocity is along the *x*-direction, is contributed from the faces normal to the *z*-axis, evaluated in equation (3.4), with the energy-release rate obtained as

3.4 $$\begin{array}{rl} \delta {\dot{\Pi }}^{H} & \;=\underset{\varepsilon \to 0}{\lim}\int_{S_{{\;}_{d}}}\sigma_{ij}{\dot{u}}_{i}n_{j}\;\text{d}x=\underset{\varepsilon \to 0}{\lim}\int_{-1/\varepsilon^{1/n}}^{1/\varepsilon^{1/n}}\sigma_{yz}(x,0,t)n_{z}b_{y}\dot{l}(\delta (x-l(t))\;\text{d}x\\ & \;=\dot{l}b_{y}\sigma_{yz}(l(t),0,t)=\dot{l}b_{y}^{2}\mu \frac{\ddot{l}}{4\pi c_{2}^{2}\gamma^{3}}\ln\varepsilon , \end{array}$$

where use was made of equation (2.5*a*). There is no energy released due to the 1/*r* terms in the field quantities corresponding to steady-state motion, since the energy received in the core by the radiation already emitted by the dislocation in the infinite past time is equal to that radiated at the current time. There is no characteristic length scale. If the motion is not at constant velocity, the two energy fluxes (received and emitted) are not equal, and the ‘dislocation is haunted by its past’ [1]. In non-uniform motion, this difference in energy fluxes is obtained in equation (3.4). Naturally, for the stress being the applied $\sigma_{yz}^{\text{appl}}$, the last two terms in equation (3.4) provide an equation of motion for the dislocation, relating the loading to the acceleration.

Finally, equations (3.1) with (3.3) and (3.4) make the connection of the dynamic *J* integral to the energy-release rate, for the dislocation as

3.5 $$\begin{array}{rl} J_{k}^{\text{dyn}} & \;=\int_{S_{d}}\{(W-T)n_{k}-\sigma_{ij}u_{j,k}n_{i}\}\;\text{d}S-\left(-\int_{V_{d}}\frac{\partial }{\partial t}(\rho {\dot{u}}_{j}u_{j,k})\;\text{d}V\right)=\frac{\dot{E}}{{\dot{l}}_{k}}\\ & \;=\mu b_{y}^{2}\frac{{\ddot{l}}_{k}}{4\pi c_{2}^{2}\gamma^{3}}\ln\varepsilon , \end{array}$$

with *k* = 1, the motion being along the *x*-direction. Equation (3.5) demonstrates the physical meaning of the dynamic *J* integral enclosing a moving dislocation, as equal to the difference of the rate of change of quasi-momentum in the volume enclosing the defect minus the flux of it through the surface, which are not balanced in non-uniform motion, but give a negative quasi-force. Through Noether's theorem, connecting the variation of the Lagrangian to the variation of the Hamiltonian, this quasi-force is associated with the energy release rate (in equation (3.1)). The last term in (3.5) yields exactly the same value for the effective mass (the coefficient of the acceleration) as the one obtained from the loss of quasi-momentum across scales in equation (3.7), thus asserting the validity of its definition, and its physical meaning.

## 4. Conclusion

The loss of pseudo-momentum (or quasi-momentum) across scales (ε to ε^2^) induces an effective mass to a non-uniformly moving dislocation associated with the acceleration. Through Noether's theorem, in accelerating dislocations the loss in the pseudo-momentum is related to the loss in energy flux, showing that both loss in quasi-momentum and energy-release entail the same effective mass. This establishes a physically sound definition for the effective mass, giving it meaning both through momentum and energy, as was suggested by Eshelby [1]. Pseudo-momentum has been widely considered in fluids, e.g. Ben Amar and Rice [18] showed the analogy in the application of the *J* integral between a screw dislocation and a vortex line, while the pseudo-momentum of a disturbance has been used in obtaining instabilities in sheared fluid interfaces (e.g. Eaves and Balmforth [19]). Thus, the obtained result on loss of pseudo-momentum across scales can have wider applications in a micromechanics modeling of continua.

## Data Availability

This article has no additional data.
